# Genomic and functional characterization of novel therapeutic lytic bacteriophages targeting multidrug-resistant *Enterobacter cloacae*

**DOI:** 10.3389/fmicb.2026.1838809

**Published:** 2026-09-01

**Authors:** Mohammad S. A. Al-Obaidi, Mushtak T. S. Al-Ouqaili, Farah Al-Marzooq

**Affiliations:** 1 Anbar Health Directorate, Department of Inspection, Anbar Governorate, Ramadi, Iraq; 2 Department of Microbiology, College of Medicine, University of Anbar, Anbar Governorate, Ramadi, Iraq; 3 Department of Microbiology and Immunology, College of Medicine and Health Sciences, United Arab Emirates University, Al-Ain, United Arab Emirates

**Keywords:** bacteriophage, *Enterobacter cloacae*, multidrug-resistant, phage therapy, whole genome sequencing

## Abstract

The alarming rates at which extensively drug-resistant (XDR) and pandrug-resistant (PDR) *Enterobacter cloacae* in hospitals are increasing has begun to severely limit treatment options, and thus the urgency for alternative interventions, including bacteriophage therapy. The purpose of the study was to isolate and molecularly characterize phages that can infect *E. cloacae*, and, furthermore, to assess the antimicrobial efficacy of the four novel lytic bacteriophages (MMRP1, MMRP2, MMRP3, and MMRP4) against antimicrobial-resistant *E. cloacae* isolates and to evaluate their potential as alternative therapeutic strategies. These novel phages were characterized by plaque morphology, transmission electron microscopy (TEM), host range testing, thermal and chloroform stability assays, bacterial reduction assays, and whole-genome sequencing (WGS). Among 27 clinical isolates, MDR, XDR, and PDR phenotypes were observed in 20 (74.1%), six (22.2%), and one (3.7%) isolates, respectively. All four phages produced clear lytic plaques (0.5–3.0 mm) with titers reaching up to 6 × 10^10^ PFU/mL, and the phage cocktail lysed 81.4% (22 of 27 isolates) of clinical isolates with high host specificity. TEM revealed that all four *E. cloacae*-infecting phages (MMRP1, MMRP2, MMRP3, and MMRP4) belong to the class *Caudoviricetes*, exhibiting icosahedral capsids, tailed morphology, and double-stranded DNA genomes, consistent with current ICTV classification criteria. Whole genome sequencing and comparative phylogenetic analysis further resolved the taxonomic placement of these phages at the family level, positioning MMRP1 within the family *Demerecviridae* and MMRP4 within the family *Straboviridae*. All phages were stable from −20 to 40 °C and were unaffected by exposure to chloroform. Phage cocktail reduced bacterial OD₆₀₀ to ≤ 0.3 within 4 h in the bacterial reduction test. WGS revealed large circular dsDNA genomes of ~132 kbp (MMRP1) and ~149 kbp (MMRP4), GC content of 38%, and modular architectures encoding structural, lytic, and replication gene modules. The most striking and highlighted suggestion that *in vitro* evaluation of MMRP1 and MMRP4 are highly recommended to more deeper future experimental studies to combat MDR *E. cloacae* nosocomial infections supported by genomic foundation and eventually, the possibility to be suitable for phage-engineering applications in clinical settings.

## 1 Introduction

Antimicrobial resistance (AMR) has become a major health issue that is estimated to be responsible for approximately 1.91 million deaths globally in 2050 due to the current trend in AMR infections and the growing number of associated deaths, even considering the 8.22 million deaths already linked to AMR infections (Tang et al., 2023).

Antimicrobial resistance can be defined as the ability of microorganisms such as bacteria, viruses, fungi, and parasites to develop resistance to one or more antimicrobial agents that would otherwise inhibit their growth or kill them. Adaptive resistance mechanisms allow pathogenic microorganisms to survive within a host environment despite therapeutic intervention (Solanki et al., 2024). The World Health Organization (WHO) considers AMR one of the ten major threats to global health. Clinical outcomes, duration of hospital stay, mortality, and healthcare expenses are consistently associated with AMR infections across all regions (Siaba et al., 2025).

Carbapenem-resistant and third-generation cephalosporin-resistant *E. cloacae* are members of the so-called ESKAPE group, a group of pathogens that was prioritized by the WHO because of their ability to develop resistance and cause severe infections (Fu et al., 2025). *E. cloacae* possesses numerous virulence factors, such as biofilm formation and capsule production, and an exceptional ability to gain resistance genes via horizontal gene transfer, as well as having an intrinsic resistance to numerous antibiotic classes, thus contributing to its pathogenicity and antimicrobial resistance (Sekhi and Jaber, 2025). *E. cloacae* needs particular consideration due to its inherent resistance to ampicillin, broad-spectrum cephalosporins, and its production of extended-spectrum *β*-lactamase (ESBL) and carbapenemase enzyme levels. Moreover, it carries aminoglycoside and fluoroquinolone resistance genes on plasmids, thereby significantly reducing the effectiveness of other antimicrobial agents (Miller and Arias, 2024).

The incidence of multidrug-resistant (MDR), extensively drug-resistant (XDR), and pandrug-resistant (PDR) *E. cloacae* bacteria in hospital-acquired infections, including bloodstream infections (CLABSI), catheter-associated urinary tract infections (CAUTI), surgical site infections (SSI), hospital-acquired pneumonia (HAP), and ventilator-associated pneumonia (VAP), has, somewhat alarmingly, been increasing rapidly. This rising prevalence of antimicrobial resistance has driven a transition to non-antibiotic treatment methods such as probiotic treatments, nanomaterial-based therapies, and genome-editing processes, including CRISPR and bacteriophage therapy, which have gained widespread popularity in recent years (Al-Ouqaili et al., 2025; Mohammadzadeh et al., 2025). Bacteriophages employed as therapeutic agents have several benefits compared to conventional antibiotics, namely high specificity (sparing the normal microbiota and eukaryotic cells), low toxicity, self-replication, and flexibility to target bacterial pathogens. On the other hand, failure in phage treatment therapy can also be attributed to various factors, such as divergence of target strain, phage stability, and the lack of adherence of cocktails to clinical strains that were ultimately treated compared to the phage infectivity against the bacterial isolates on which the cocktails were developed (Subedi et al., 2025).

Bacteriophages can be classified at two hierarchical levels. Morphological characterization via transmission electron microscopy (TEM) is traditionally employed to assign phages to established classes (*Caudoviricetes*, *Leviviricetes*, *Faserviricetes*, *Tectiliviricetes*, and the newly erected *Ainoaviricetes*). However, family-level designation is now primarily based on whole-genome sequencing (WGS) coupled with comparative phylogenetic analysis. Within *Caudoviricetes*, 14 families have been formally assigned to four orders, with 33 additional families established but pending ordinal placement. These taxa are distinguished according to host range and molecular characteristics. Although the terms myovirus, podovirus, and siphovirus retain utility as non-taxonomic morphological descriptors, this classification adheres to the revised ICTV criteria (2022), which has dropped morphology-based classification in favor of genome-informed phylogenetic frameworks (Turner et al., 2023).

The isolation and molecular characterization of *Enterobacter* bacteriophages have rarely been studied in the Arab countries, including Iraq. This work was undertaken to isolate and molecularly characterize environmental *E. cloacae* phages and use clinically resistant *E. cloacae* isolates as host strains. Additionally, the *in vitro* antimicrobial activity of the phages identified against multidrug-resistant *E. cloacae* isolates was evaluated to assess their potential as alternative treatment options in future clinical practice.

## 2 Materials and methods

### 2.1 Identification and antimicrobial susceptibility testing of *Enterobacter cloacae* isolates

A total of 27 clinically resistant *E. cloacae* isolates, used as bacterial hosts for bacteriophage isolation, were collected between May 20, 2024, and September 1, 2025. All isolates were collected according to the following inclusion criteria: antimicrobial-resistant *E. cloacae* nosocomial strains recovered from patients aged between 1 month and 85 years who had been admitted to the intensive care unit (ICU), had undergone surgery, had respiratory disease, or had suffered from burns at the participating hospitals. These isolates were collected from six major hospitals across Iraq, namely Ramadi Teaching Hospital, Ramadi Maternity and Children Hospital, Al Fallujah Teaching Hospital, Ghazi Al-Hariri Hospital, Baghdad Burns Specialist Hospital, and Baghdad Teaching Hospital. Sewage samples used for phage isolation were collected from the main sewage reservoir of Ramadi Teaching Hospital.

Different clinical specimens were collected, including catheterized urine, blood, wound swabs, endotracheal aspirate, nasogastric wash, and burn wound swabs, which were processed and cultured in the microbiology laboratories of the same hospitals. All bacteriological examinations were performed using the BacT/ALERT 3D system and VITEK2 for the detection and identification of pathogenic microbes, with complete demographic and clinical data available. Patients were excluded if their records lacked essential demographic or microbiological information, or if their isolates were not resistant *E. cloacae*.

The identification of *E. cloacae* isolates was confirmed using the BacT/ALERT 3D and VITEK2 automated identification systems. In total, of the 562 patient specimens initially screened, 27 *Enterobacter cloacae* resistant isolates were selected for phage propagation and isolation following the application of specific exclusion criteria. Any isolates not identified as *E. cloacae* were excluded, as were antimicrobial-susceptible *E. cloacae* isolates. The MICs of the selected clinical isolates were evaluated against 19 antibiotics, each with distinct mechanisms of action, using the VITEK2 AST-GN card in accordance with the manufacturer’s instructions to ensure accuracy (Csiki-Fejer et al., 2025). Based on antibiotic susceptibility profiles, the strains were classified as multidrug-resistant (MDR) if resistant to at least one agent in three or more antimicrobial categories, extensively drug-resistant (XDR) if resistant to at least one agent in all but two or fewer antimicrobial categories, and pan-drug-resistant (PDR) if resistant to all agents in all antimicrobial categories (Jwair et al., 2023).

Written informed consent was obtained from all participants in the study, including patients and/or their parents, in accordance with the questionnaire approved by the Medical Ethics Committee of the University of Anbar, Iraq (approval number 51, April 3, 2024). The study was conducted in accordance with the 2013 Declaration of Helsinki. Phage experiments and bacteriological analyses were performed in accordance with standard biosafety-level protocols.

### 2.2 Phage isolation

Active *E. cloacae* phages were isolated from sewage from the Ramadi Teaching Hospital (west of Iraq) using an enrichment method (Manohar et al., 2019). Within 1 h of sewage collection, 10 mL of sewage was centrifuged at 5000 × g for 15 min at room temperature, with the supernatant collected in a sterile test tube. Ten previously isolated *E. cloacae* strains (with IDs E1, E2, E4, E5, E8, E9, E15, E19, E22 and E24) were cultured in Tryptic Soy Broth and subsequently used as hosts for bacteriophage isolation. Briefly, 1 mL of exponentially grown bacterial culture (0.1 mL of each isolate) (OD_600_ nm = 0.6), 10 mL of TSB broth, 15 mL of phage-containing sewage sample, and 2 mL of lambda buffer or SM buffer, with the chemical structure of sodium chloride 100 mM, magnesium sulfate 8 Mm, and Tris–HCL 50 mM (PH 7.5) were added to 30 mL glass tubes. Two gram of MgCl_2_ crystals were then added to enhance phage adsorption. The mixture was incubated at 37 °C for 24 h in a shaking incubator to enrich the phages against the host bacterium. After centrifugation at 12,000 × g and filtration through a 0.22-μm pore-sized sterile nitrocellulose syringe filter, the mixture was centrifuged and filtered to remove other contaminants besides the phages. The filtrate was stored at 4 °C for further investigation (Hyman, 2019).

#### 2.2.1 Spot test method

The active lytic phages in the freshly collected lysates were tested using a spot test. An aliquot of 10 μL lysate was placed on the newly prepared bacterial lawn of the MDR *E. cloacae* isolate, standardized to 0.5 McFarland turbidity at the log growth phase, and mixed with molten Tryptic Soy agar at 45 °C. The plates were incubated overnight at 37 °C, after air-drying the drops for 10–20 min at room temperature. The formation of transparent lytic spots on the bacterial lawn surface was indicative of the presence of the bacteriophage and its lytic nature (Mutai et al., 2022).

After identification, *E. cloacae*-specific lytic phages were picked using a sterile loop and transferred into 2 mL SM buffer in sterile 5 mL Eppendorf tubes. To eliminate any residual bacteria, the mixture was either gently shaken for 5 min or vortexed for 20 s, then centrifuged at 4000 × *g* for 10 min to pellet bacterial cell debris, and finally filtered through a sterile 0.22 μm nitrocellulose syringe filter. The filtered supernatant containing isolated phages was stored at 4 °C and transferred to 1.5 mL sterile Eppendorf tubes for use in the subsequent procedure. This supernatant is referred to as a transient phage stock suspension (Daubie et al., 2022).

#### 2.2.2 Top-layer agar plaque assay for phage characterization and titration

Phage lysates were serially diluted tenfold in SM buffer across five dilution points (10^−1^, 10^−3^, 10^−5^, 10^−7^, and 10^−10^). At each dilution, 100 μL of the lysate was mixed with 200 μL of a multidrug-resistant *E. cloacae* isolate selected from among ten candidate host strains previously employed for phage isolation and standardized to the logarithmic growth phase at an optical density of OD_600_ = 0.6. This bacterial suspension was then mixed with 2.5 mL of molten top agar (0.7%) nutrient medium, maintained at 45 °C. The mixture was then poured onto solidified nutrient agar (1.5% agar) and incubated for 24 h at 37 °C. Primary clear plaques were classified according to their size, shape, and clarity. A double-layer agar method was subsequently used to obtain purified phages (Asif et al., 2021).

#### 2.2.3 Single-phage plaque purification and preparation of high-titer stock

A single clear plaque was selected and purified through multiple rounds of plaque isolation. This process was repeated until a consistent single-plaque morphology was achieved, ensuring phage purity. The purified phage was then propagated, and the primary phage titer was determined. The titer was expressed as plaque-forming units per milliliter (PFU/mL). High-titer phage stocks (about ≥10^15^ PFU/mL) were prepared and subsequently used for DNA extraction.

##### 2.2.3.1 Equation for phage titer calculation

$$\text{Phage titer}\;(\mathrm{PFU}/\mathrm{mL})=\frac{\text{Number of plaques}\times \text{Dilution factor}}{\text{Volume plated}\;(\mathrm{mL})}$$

#### 2.2.4 Examination of phage morphology by transmission electron microscopy (TEM)

Morphological examination of purified phage particles was performed via TEM. Ten microliters of phage solution were placed on a carbon support film on a copper mesh grid surface and negatively stained with UranyLess stain; staining was carried out in strict accordance with the manufacturer’s guidelines (em-grade) to ensure optimal clarity and reproducibility. The grid was examined using a scanning-transmission electron microscopy (STEM) instrument (Quattro S, Thermo Scientific, Germany) at an operating voltage of 20 kV. All four isolated phages were initially classified based on morphological characteristics, and this classification was subsequently reinforced for MMRP1 and MMRP4 using whole-genome sequencing (WGS) data, in accordance with the International Committee on Taxonomy of Viruses (Kąkol et al., 2023).

#### 2.2.5 Thermal and chloroform stability of isolated *E. cloacae* phages

Four purified isolated phages were subjected to different physicochemical conditions, temperature and chloroform stability. To determine thermal stability, the phage preparations were incubated in SM buffer at different temperatures (−20, 4, 37, 50, and 75 °C) for 1 h, after which the titer of the virus was assessed. To determine chloroform stability, 2 mL (1 × 10^5^ PFU) of the phage was mixed with 0.2 mL of chloroform, and the phage was collected and titered after 1 h of incubation at 37 °C.

#### 2.2.6 Host range of the bacteriophage

The phage host range was evaluated using the spot method. Briefly, 27 MDR, XDR, and PDR clinical and nosocomial isolates of *E. cloacae* were used, in addition to international standard quality control isolates from the American Type Culture Collection: *Pseudomonas aeruginosa* (ATCC 27853), *Escherichia coli* (ATCC 25922), *Streptococcus pyogenes* (ATCC 19615), and *Acinetobacter baumannii* (ATCC 19606). These were cultured until they reached the exponential growth phase (OD_600_ = 0.6). Thereafter, 100 μL of each culture was mixed with 3 mL of top agar containing 10 mM MgCl_2_ and poured onto a 2% nutrient agar plate. After solidification, a single droplet (10 μL) from each isolated single- and cocktail-phage at dilution (10^−5^) was applied to the agar. The droplets were allowed to dry for 10 min, the plates were incubated at 37 °C, and phage clearance was assessed after 18–24 h.

### 2.3 Bacterial reduction assay

One mL of a fresh culture of *E. cloacae* (OD_600_ = 0.1, 1 × 10^7^ CFU/mL) was inoculated into two separate 30 mL glass tubes containing 25 mL TSB. One flask was inoculated with 0.2 mL of a high-titer phage lysate (1,500,000 PFU/mL, MOI = 15), and the other, without phage, was used as a negative control. The cultures were incubated at 35 °C and 160 rpm. The optical density (OD_600_) of each sample was measured at 30 min intervals for 5 h. The experiment was performed in two independent replicates, and results are presented as mean ± standard deviation (SD).

### 2.4 Quality control of phage DNA to improve phage DNA extraction

To confirm the sterility of the lysate, 10 μL of lysate was spread onto nutrient agar plates without host bacteria and incubated at 37 °C for 24 h. Lysate sterility was confirmed by the absence of observable bacterial growth. Free nucleic acids were removed by sequential treatment with DNase I (1 U/μL) and RNase A (10 μg/mL), as applied prior to capsid disruption during the DNA extraction steps (Zhu et al., 2025).

### 2.5 Precipitation of isolated *E. cloacae* phage using a 20% PEG 2.5 M NaCl solution

The phage lysate was mixed with a premade solution of 20% (w/v) PEG and 2.5 M NaCl at a 1:5 (v/v) ratio, and incubated at 4 °C for 12 h to encourage phage precipitation. High-speed microcentrifugation was then performed on the mixture at 12,000 × g at 5 °C using a refrigerated high-speed microcentrifuge (Eppendorf, Germany), with a concentration efficiency of approximately 80%. The resulting pellet was resuspended in 1 mL of TBS buffer (20 M Tris, 150 M NaCl; pH 7.6) at a 1: 15 ratio of the original phage lysate, with the supernatant then transferred to clean tubes following centrifugation and phage DNA extraction protocols, as well as performing phage morphology imaging via TEM (Kılıç et al., 2025).

### 2.6 Phage DNA extraction

To extract phage DNA, the SaMag-12 automated nucleic acid extraction system, and the SaMag Viral Nucleic Acids Extraction Kit (Sacace Biotechnologies, Italy) were used. The extraction procedure consisted of the following stages: preliminary lysis of the viral particle, binding of nucleic acid to magnetic beads, washing, and elution of purified DNA.

The concentration of the extracted DNA was determined using a Qubit fluorometer (Invitrogen). Sample purity was evaluated using a NanoDrop One instrument (Thermo Scientific, USA). DNA integrity was confirmed via agarose gel electrophoresis performed on 1% (w/v) agarose gels at 150 V for 50 min (Supplementary Figure S3). Following quality assessment, pure and intact DNA samples were preserved at −60 °C in a deep freezer (Arctiko Integraline, Marshall Scientific, United States) until DNA sequencing analysis (Li et al., 2023).

### 2.7 Sequencing and bioinformatic analysis of phage genomes

Whole-genome sequencing (WGS) was performed by BGI (Shenzhen, China) using the DNBSEQ platform after the construction of high-quality ssCir DNA libraries. DNA fragmentation and library preparation were performed by randomly fragmenting DNA with a Covaris instrument, and fragmented genomic DNA was selected using the AMPure XP-Medium Kit to an average size of 200–400 bp. Fragments were end-repaired and then adenylated at the 3′ end. Adaptors were ligated to the ends of these 3’adenylated fragments to amplify the fragments with adaptors. PCR products were subsequently purified using the AMPure XP-Medium Kit. Double-stranded PCR products were heat-denatured and circularized using the splint oligo, and the resulting single-stranded circular DNA (ssCir DNA) used to construct the final library. The library was verified using quality control (QC).

Qualified libraries were subsequently sequenced using the DNBSEQ platform (BGI, Shenzhen, China). DNA nanoballs (DNBs) formed from ssCir DNA molecules that contained more than 300 copies through rolling-circle replication were loaded onto patterned nanoarrays using high-density DNA nanochip technology, and paired-end reads were obtained using combinatorial probe-anchor synthesis (cPAS) chemistry.

Raw sequencing reads were first inspected using FastQC v0.12.1. Low-quality bases and adapter sequences were removed using Trimmomatic v0.39 with the following parameters: SLIDINGWINDOW:4:20 and MINLEN:50. The filtered reads were then assembled *de novo* using Unicycler v0.4.8 with default parameters. Genome completeness and potential host contamination were assessed using CheckV v1.0.3. Genome annotation was performed using Pharokka, and coding sequence prediction was carried out using PHANOTATE v1.6.7. The Pharokka workflow also included tRNAscan-SE, MinCED, Aragorn, MMseqs2, PHROG-based annotation, VFDB screening, CARD screening, and terminase large-subunit detection. A total of 58 CDSs, 0 predicted AMR genes, and 0 predicted virulence factors were identified. Functional annotation showed 32 CDSs of unknown function, together with predicted genes related to tail structure, DNA/RNA metabolism, head and packaging, connector proteins, lysis, other functions, and auxiliary/host takeover functions.

Phylogenetic analysis was performed using the complete genome nucleotide sequences of bacteriophages MMRP1 and MMRP4 together with closely related reference phage genomes retrieved from the NCBI GenBank database based on BLASTn similarity searches. Multiple sequence alignment was carried out using MAFFT v7.520 with default parameters. Maximum-likelihood phylogenetic trees were reconstructed using IQ-TREE 2 (v2.2), and the optimal nucleotide substitution model was automatically selected using ModelFinder according to the Bayesian Information Criterion (BIC). Branch support was assessed by 1,000 ultrafast bootstrap replicates. Trees were midpoint-rooted for visualization because no appropriate outgroup genome was available. The resulting trees were visualized and annotated using the Interactive Tree of Life (iTOL v6). BLASTn and Average Nucleotide Identity (ANI) analyses were additionally performed using BioPython and custom Python scripts.

Intergenomic similarity analysis of phages MMRP1 (GenBank accession PZ534072) and MMRP4 (GenBank accession PZ534073) was performed using the Virus Intergenomic Distance Calculator (VIRIDIC) (Moraru et al., 2020) against a curated set of reference genomes retrieved from the NCBI GenBank database. Complete nucleotide sequences were aligned in a pairwise manner to compute intergenomic similarity percentages, aligned genome fractions, and genome length ratios for each genome pair. Taxonomic classification followed the demarcation criteria recommended by the International Committee on Taxonomy of Viruses (ICTV) Bacterial and Archaeal Viruses Subcommittee, whereby genome pairs sharing ≥95% nucleotide identity were assigned to the same species, and those sharing ≥70% identity were assigned to the same genus (Turner et al., 2023).

### 2.8 Statistical analysis

Data from the bacterial reduction assay and the thermal and chloroform stability assays were obtained from two independent experimental replicates and are expressed as mean ± standard deviation (SD). Due to the limited number of replicates, formal statistical significance testing was not performed; this represents a limitation of the study, and future work will incorporate a larger number of biological replicates with appropriate statistical testing to confirm the significance of observed differences.

## 3 Results

### 3.1 High prevalence of MDR and XDR phenotypes among clinical *E. cloacae* isolates

**Figure 1:**
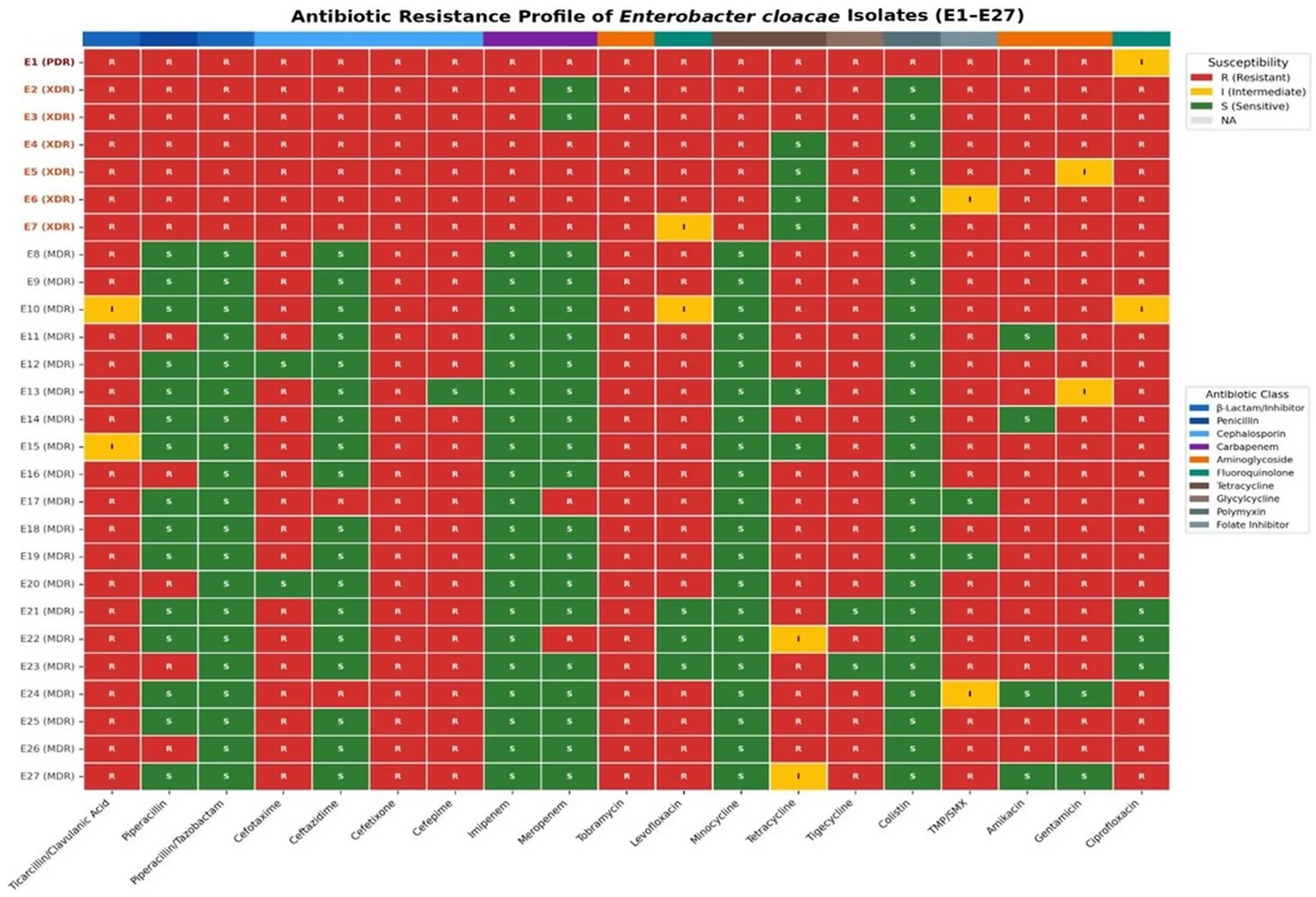
Antimicrobial susceptibility for 27 *E. cloacae* isolates against 19 antimicrobial agents across different classes, based on MIC values.

Twenty seven *E. cloacae* isolates were selected for phage recovery from sewage samples. The susceptibility profiles of these isolates revealed a predominance of MDR (20 isolates, 74.1%) and XDR (six isolates, 22.2%) phenotypes, and one PDR isolate (3.7%). All isolates were resistant to ceftriaxone and tobramycin, and colistin represented the most active antimicrobial agent *in vitro*, with a susceptibility rate of 96.3% across the tested isolates (Figure 1).

### 3.2 Four lytic phages with broad host range effectively target MDR *E. cloacae* isolates

**Figure 2:**
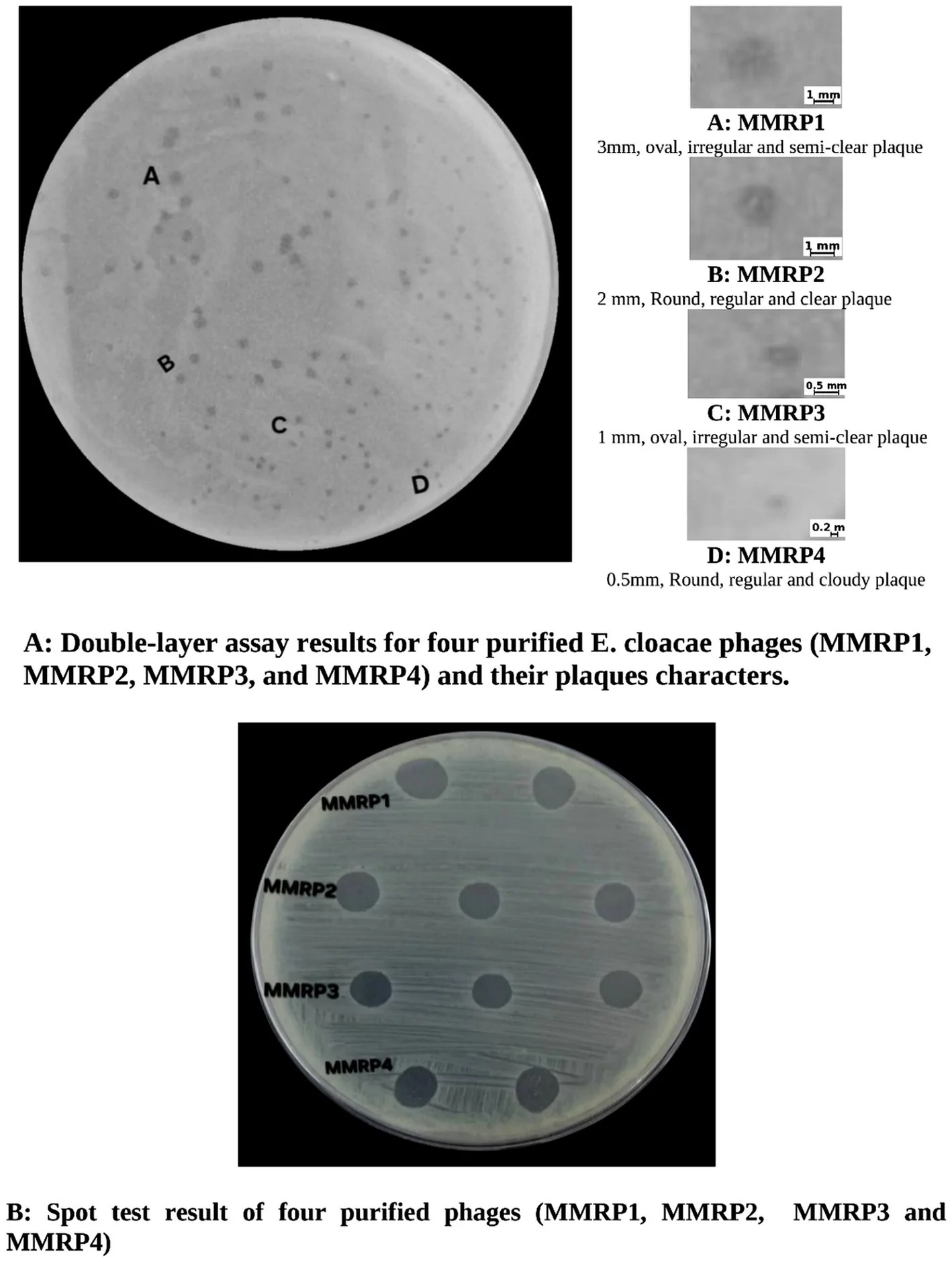
Double-layer assay **(A)** and spot test assay **(B)** results for four purified *E. cloacae* phages (MMRP1, MMRP2, MMRP3, and MMRP4).

**Figure 3:**
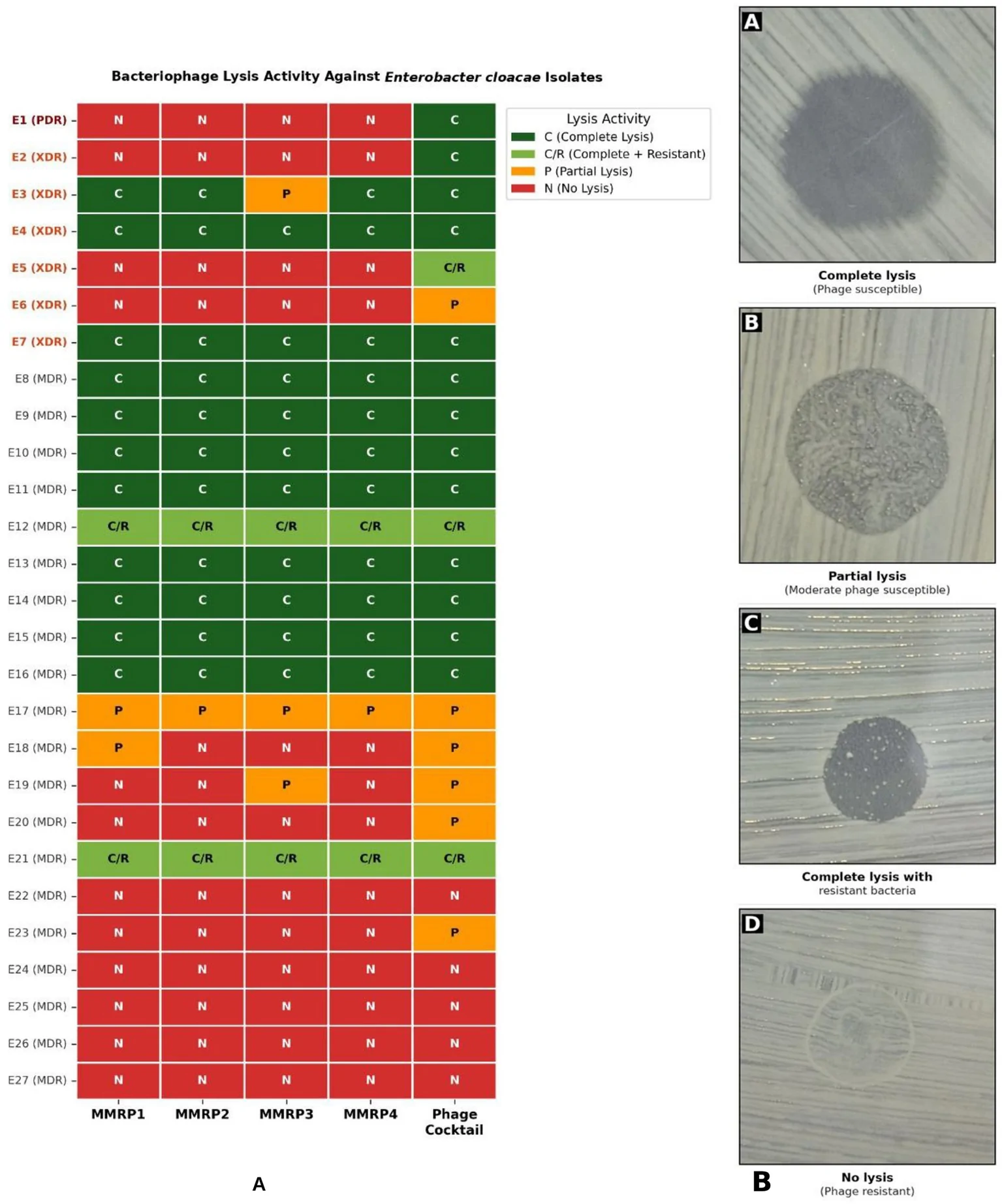
Host range test of four, and their related cocktail, isolated *E. cloacae* phages **(A)** Host range test results of four purified phages **(B)** intensity of the clearing zone was directly related to isolates’ lytic activity.

Among the 27 clinical *E. cloacae* isolates, two showed excellent lysis susceptibility and were selected as hosts for lytic phage isolation. Spot screening of three sewage water samples yielded four bacteriophage isolates capable of producing distinct lytic plaques. Assessment of the plaque morphologies of the four selected phages at a dilution of 10^5^ using the primary double-layer agar method revealed well-defined, regular, and circular plaques with clear lytic zone diameters ranging from approximately 0.5–3 mm (*n* = 144) (Figures 2A,B). The phage titers of the isolated phages ranged from 1.4 × 10^8^ to 6 × 10^9^ PFU/mL, with the lowest dilution rate yielding a visible lysogenic titer of 10^−4^, suggesting that it had a strong ability to infect host cells at relatively low concentrations. Host range spectra of the 27 clinical *E. cloacae* isolates revealed that the phage cocktail could infect and lyse 81.4% (22 of 27 isolates) of the *E. cloacae* isolates. The results demonstrated that all four of the phages isolated and phage cocktails were specific to *E. cloacae* and did not affect other bacterial species (*P. aeruginosa*, *E. coli*, *S. pyogenes,* and *A. baumannii* ATCC bacterial strains). The intensity of the clearing zone was directly related to the phage’s ability to lyse the clinical isolate. Lytic activity was graded as follows: (1) complete clearing (phage susceptible), (2) clearing with a faint hazy background (moderate phage susceptibility), and (3) no clearing (phage resistant) (Figure 3).

### 3.3 TEM confirms tailed, icosahedral morphology consistent with class *Caudoviricetes*

**Figure 4:**
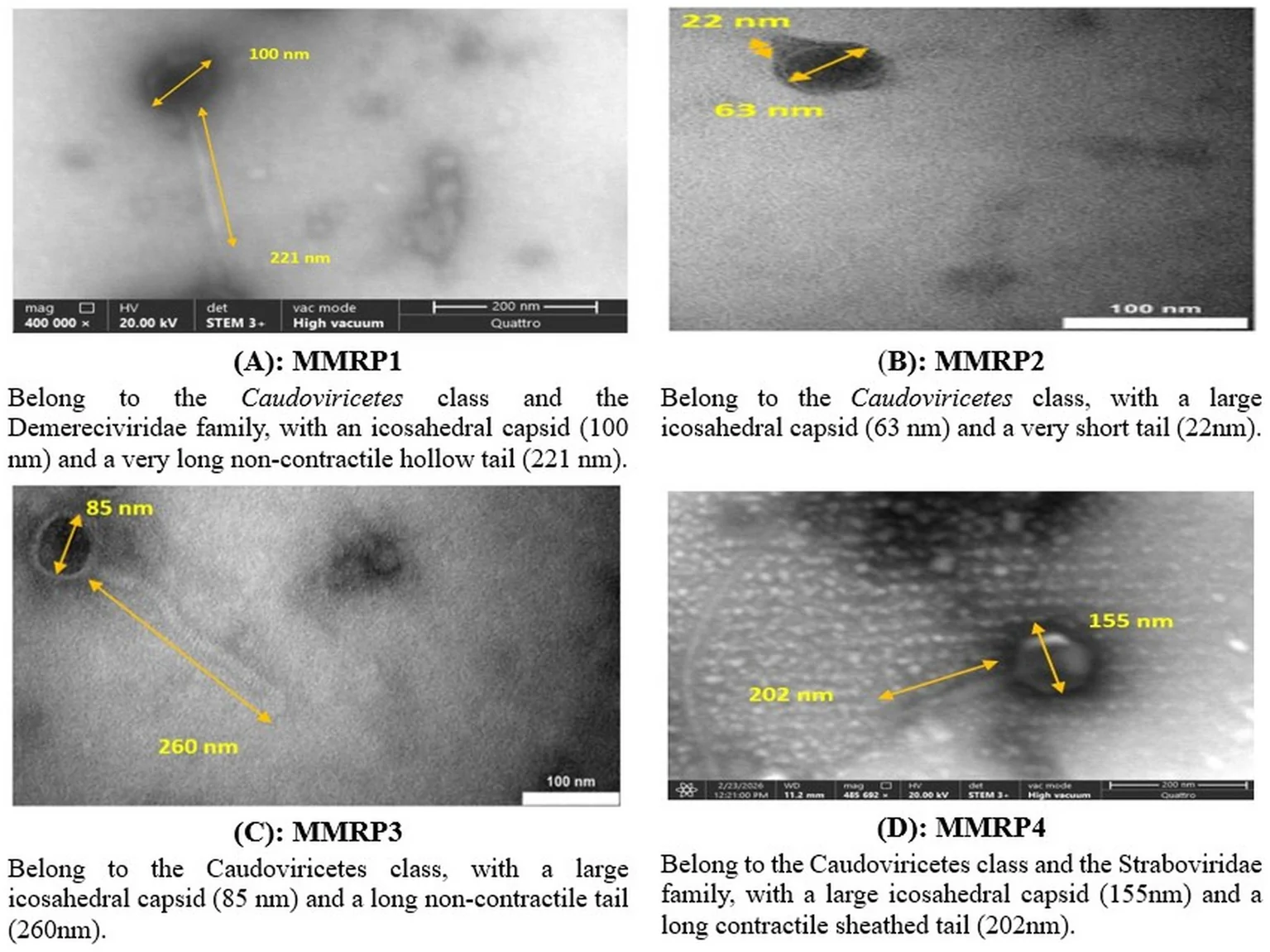
Transmission electron micrographs. Scale bars represent 100 and 200 nm, magnification 400,000x–500,000x. Three representative images **(A–D)** indicate purified four phage particles.

TEM was used to assign all four isolated *E. cloacae*-infecting phages to the class *Caudoviricetes*, characterized by icosahedral capsids, tailed, and double-stranded DNA genomes. Definitive family-level classification within *Caudoviricetes* now mandates whole-genome sequencing and comparative phylogenetic analysis, which were subsequently performed for MMRP1 and MMRP4, and which were classified as being in the evolutionary *Demereciviridae* and *Straboviridae* families, respectively, as consistent with current ICTV classification criteria (2022) (Figure 4).

### 3.4 Phage cocktail significantly reduces bacterial growth within 3.5 h

**Figure 5:**
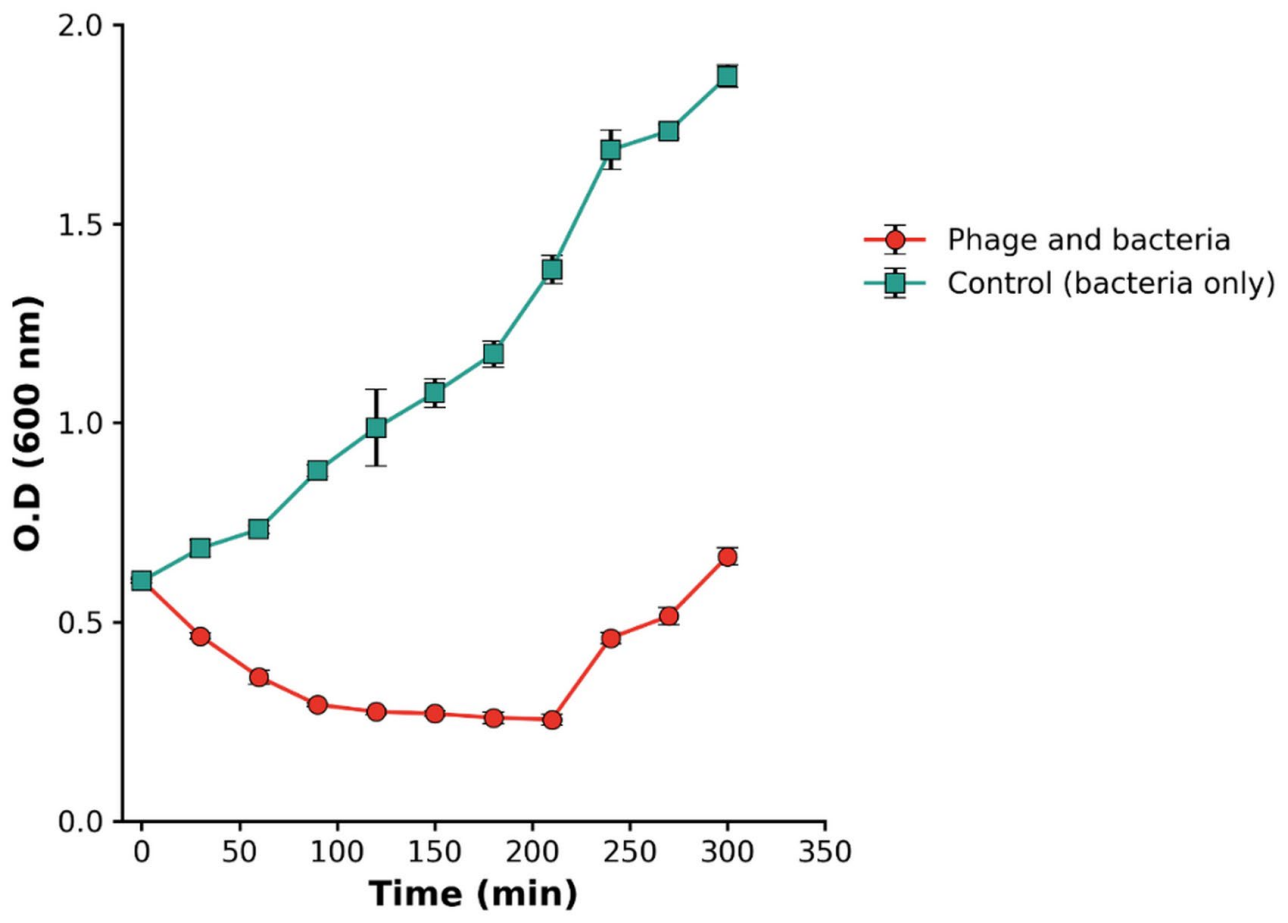
Bacterial reduction assay showing OD_600_ of phage-treated versus control *E. cloacae* cultures over 300 min. Phage treatment significantly reduced bacterial growth from 30 min onward (*p* < 0.05, two-way ANOVA with Šídák’s test), followed by regrowth after ~210 min indicating resistant subpopulations. Data are mean ± SD (*n* = 3).

A progressive decline in OD₆₀₀ was recorded in the *E. cloacae* culture (isolate ID No. 5) treated with the four-phage cocktail, in contrast to the untreated control culture, based on two independent experiments (mean ± SD). Following phage cocktail treatment, the OD₆₀₀ decreased from 0.61 ± 0.01 to a minimum of 0.26 ± 0.01 within approximately 3.5 h of incubation, reflecting a marked reduction in bacterial growth. An increase in turbidity (OD₆₀₀) was subsequently observed after approximately 4 h of incubation, most likely due to the regrowth of phage-resistant bacterial subpopulations. In contrast, the OD₆₀₀ of the untreated control culture increased continuously, reaching 1.87 ± 0.03 after 5 h of incubation, consistent with normal, unimpeded bacterial growth (Figure 5).

### 3.5 Phage cocktail remains stable across a wide temperature range and is resistant to chloroform

**Figure 6:**
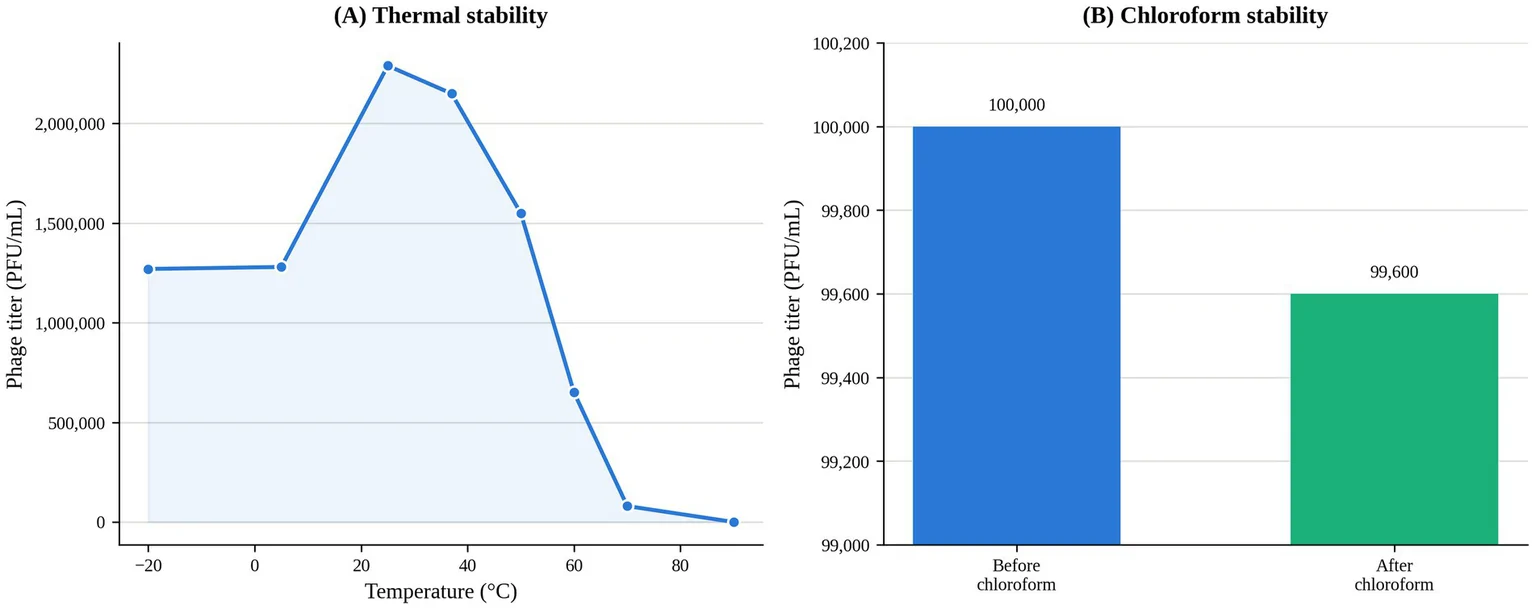
Physical stability of the phage cocktail. **(A)** Thermal stability showing phage titer (PFU/mL) across a temperature range of −20 °C to 90 °C. **(B)** Chemical stability showing phage titer before and after incubation with chloroform; no significant difference was observed between the two conditions (*p* > 0.05, paired *t*-test), confirming the stability of the phage cocktail under chloroform exposure.

The stability of the phage cocktail and its corresponding cocktails across different temperatures and chloroform concentrations was assessed. The phages were stable over a temperature range of −20 to 40 °C. However, the phage titer decreased slightly at 50 °C and dramatically at 70 °C, whilst thermal treatment at 90 °C led to the complete elimination of phage activity, consistent with protein denaturation and loss of structural integrity (Figure 6A). The phage cocktail titer showed no significant difference before (1.0 × 10^5^ PFU/mL) and after (9.96 × 10^4^ PFU/mL) chloroform treatment, indicating that the phage cocktail remained stable following exposure to chloroform (Figure 6B).

### 3.6 High-quality sequencing data support reliable genome assembly of MMRP1 and MMRP4

Whole-genome sequencing of the *E. cloacae* phages MMRP1 and MMRP4 generated 3,957,316 paired-end reads (2 × 150 bp) totaling 593.5 Mbp, with mean quality scores consistently maintained at Q35–36 across all read positions, no 3′-end degradation, and no residual adapter sequences, collectively confirming excellent base-calling accuracy and library preparation quality. Both phages exhibited identical GC content of 38%, uniformly distributed and consistent with theoretical expectations, with no low-quality reads detected and no quality-based trimming required before assembly. Genome assembly revealed that MMRP1 and MMRP4 possessed genome sizes of 131,637 bp (N50: 23,407 bp) and 148,101 bp (N50: 24,908 bp), respectively, from a combined total of 8,738,130 reads at a uniform read length of 150 bp.

### 3.7 Genomic and phylogenetic analyses reveal taxonomic novelty of MMRP1 and MMRP4

**Figure 7:**
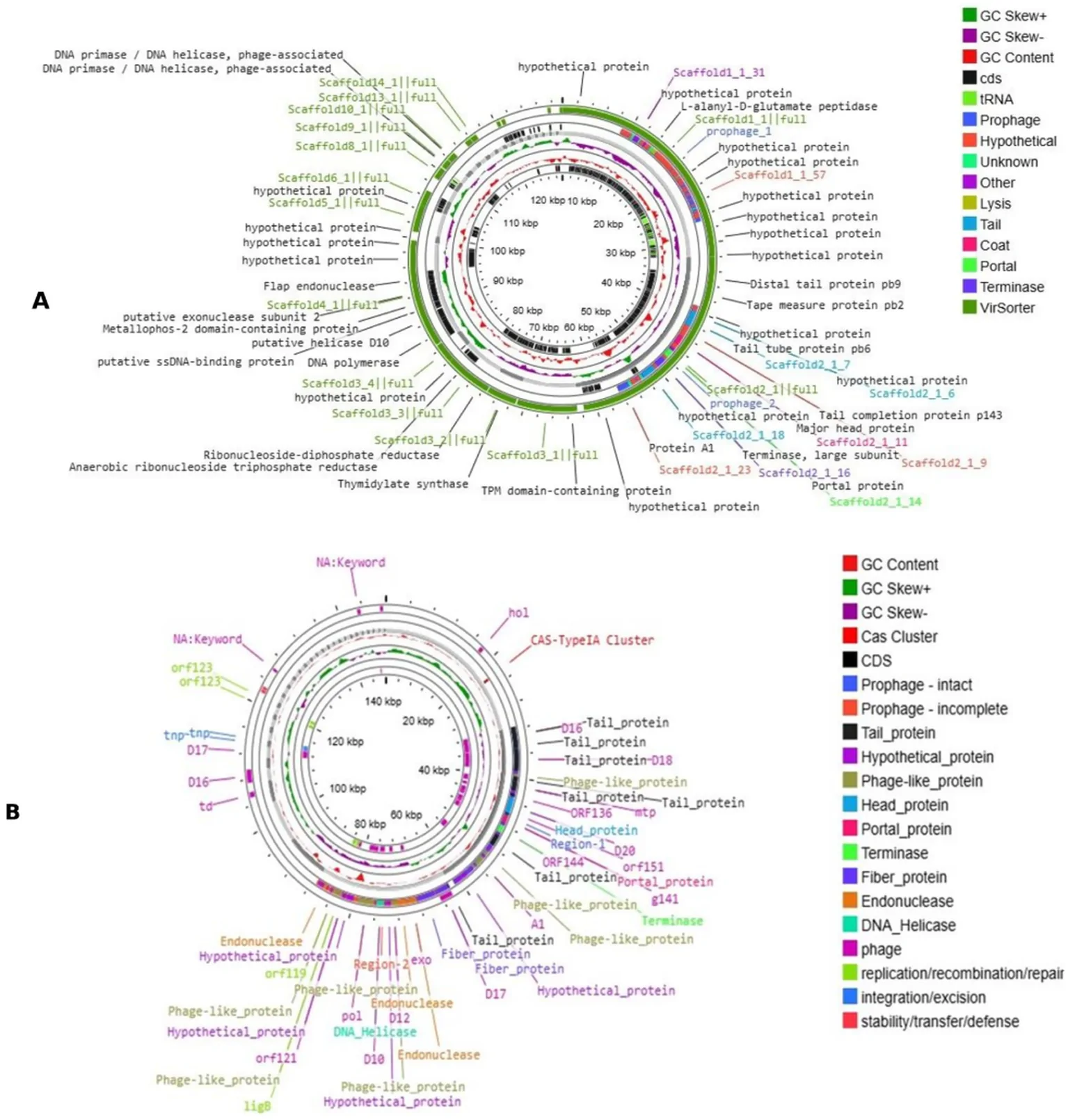
The genome maps of MMRP1 **(A)** and MMRP4 **(B)** encode a complete set of structural proteins, lytic proteins, replication enzymes, and metabolic genes, organized in a modular architecture typical of large-tailed bacteriophages.

The circular genomes of bacteriophages MMRP1 (131,637 bp; 38% GC content) and MMRP4 (148,101 bp; 38% GC content), visualized using the CG View Server, revealed well-organized genomic architectures encoding diverse arrays of functionally categorized genes distributed across both strands. While both phages share a common GC content and harbor structural proteins, replication machinery, and hypothetical proteins, MMRP1 primarily encodes lysis-associated enzymes alongside tail assembly components, whereas MMRP4 carries additional genomic features including integration/excision elements, stability/defense clusters, a CRISPR-associated Type IA (CAS-Type IA) cluster, and a putative holin (hol) gene (Supplementary Tables S1, S2). The alternating GC skew patterns observed in both genomes were consistent with bidirectional replication originating from a single replication origin, a hallmark feature of the large-tailed *Caudoviricetes* class (Figures 7A,B).

**Figure 8:**
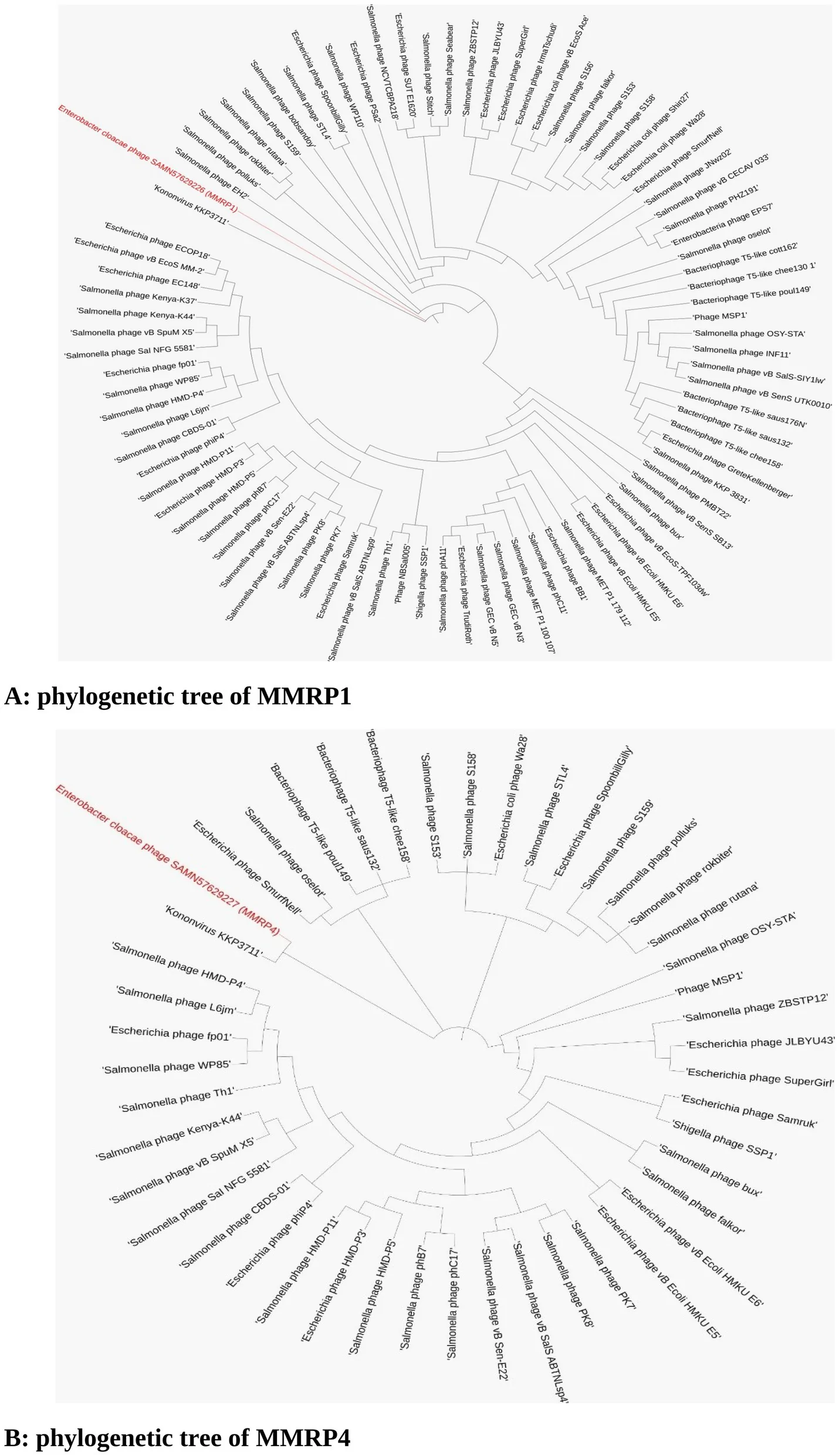
The phylogenetic analysis of MMRP1 **(A)** and MMRP4 **(B)** compared with reference phages from public databases.

Phylogenetic analysis of the assembled phage genomes, conducted by comparing their sequences against reference phage genomes retrieved from NCBI GenBank, revealed distinct yet informative evolutionary placements for both isolates. MMRP1 clustered within a well-supported clade alongside characterized *Klebsiella, Enterobacter*, and *Cronobacter* phages within the *Demereciviridae* family, positioning it within the family of large *Enterobacter* phages that share conserved structural and replication gene modules with reference strains; notably, its closest relatives included vB_VIPECLUMCO2 and *Enterobacter* phage PP579771, confirming its evolutionary relationship to therapeutically relevant phages with documented activity against MDR strains. In contrast, MMRP4 clustered within the family *Straboviridae*, demonstrating closest evolutionary relatedness to *Salmonella* and *Enterobacter* phages, with a sequence length range spanning 24,575 to 33,731 amino acids and a divergence scale of 0.06 amino acid substitutions per site. Collectively, these phylogenetic placements not only confirmed the genomic novelty of both isolates but also highlighted their potential therapeutic relevance as members of well-characterized phage lineages with established efficacy against clinically significant multidrug-resistant Enterobacteriaceae (Figures 8A,B).

VIRIDIC analysis revealed that both phages MMRP1 and MMRP4 exhibited their highest intergenomic similarity with Enterobacter phage KKP_3,711 (GenBank accession PP579741.1), sharing 86.1 and 82.2% nucleotide identity of the MMRP1 and MMRP4, respectively (Supplementary Figures S1, S2). Both values exceeded the ICTV genus-demarcation threshold (≥70%) while remaining below the species threshold (≥95%), indicating that MMRP1 and MMRP4 represent two genetically distinct, novel species within the same proposed genus as KKP-3711. In contrast, both phages shared markedly lower similarity (28.8–39.8%) with a second cluster of 15 reference genomes, which themselves formed a tightly related group sharing 79.3–100% identity among one another, confirming that MMRP1 and MMRP4 are genetically unrelated to this cluster at the genus level.

Further, comparative genomic analysis revealed considerable genome divergence, as the assembled genome length of phage MMRP1 was 131,637 bp compared with 113,711 bp for the closest reference phage, indicating substantial genomic variation beyond nucleotide substitutions alone. The BLAST alignment coverage also demonstrated discontinuous matching regions across the genome, further supporting genomic distinctiveness. Furthermore, complete genome sequences of the bacteriophages MMRP1 and MMRP4 have been deposited in the NCBI database under BioSample accession numbers SAMN57629226 and SAMN57629227, respectively, under BioProject PRJNA1460573. Furthermore, the complete genome sequences of *Enterobacter* phages MMRP1 and MMRP4 have been made publicly available in GenBank with accession numbers PZ534072 and PZ534073, respectively.

## 4 Discussion

*Enterobacter cloacae* is a common pathogen in healthcare settings and is responsible for different nosocomial infections such as pneumonia, urinary tract infections, and septicemia. The increasing incidence of multidrug resistance, particularly to carbapenem antibiotics, has become a major clinical challenge in Iraq (Shakir et al., 2025). Antibiotic susceptibility testing of the 27 *E. cloacae* isolates revealed the emergence of high rates of MDR, XDR, and PDR patterns compared to previous studies conducted in Iraq (Alzaidi and Mohammed, 2022). This underlines the need for alternative forms of therapy against MDR, XDR, and PDR nosocomial *E. cloacae* as forms of effective clinical management. Bacteriophages represent promising candidates for various clinical applications, including phage therapy, biological control, and decontamination (Victoria-Blanco et al., 2024).

In the present study, four bacteriophages exhibiting independent lytic activity against pathogenic *E. cloacae* strains with MDR, XDR, and PDR phenotypes were characterized according to their phenotypic and morphological properties, supported by genomic analysis, which was subsequently performed on two selected phages (MMRP1 and MMRP4) to further elucidate their genetic architecture and taxonomic identity. The four phages were highly lytic, as indicated by the appearance of purposely clear plaques on *E. cloacae* bacterial lawns and spot tests, which are characteristic signals of effective host cell lysis. These findings align with those of Gonzalez-Gomez et al. (2024). Over the past few years, the successful isolation and characterization of bacteriophages specific to *E. cloacae*, mostly of wastewater origin, have been reported in the literature due to its high enterobacterial load, which makes it an attractive host for phage prospecting (Hyman, 2019).

Various researchers have previously documented the presence of different sizes of plaques, such as punctuate (less than 1 mm) to a size of 5 mm in diameter (Asif et al., 2021). The same trend was observed with our two-layer findings, which indicated that plaque diameter had a range of 0.5–2.5 mm for all four phages. Plaques were clear with defined edges, consistent with a lytic phenotype that improves their use for therapeutic purposes.

The four *Enterobacter cloacae* bacteriophages (MMRP1, MMRP2, MMRP3, and MMRP4) characterized, both individually and as a combined phage cocktail, displayed a broad lytic spectrum against clinical *E. cloacae* isolates. Notably, the phage cocktail demonstrated potent lytic activity against 22 of the 27 isolates (81.4%) 22 of the 27 isolates, in accordance with the results reported by Subedi et al., in which a phage cocktail achieved an 88% host range coverage against *E. cloacae* complex (ECC) isolates (Subedi et al., 2025). In contrast, all individual phages and phage cocktails exhibited a narrow host range against the tested ATCC bacterial strains, confirming their high specificity for the target host, consistent with previous studies (Manohar et al., 2019; Mutai et al., 2022). This study disagrees with that of El-Safty et al., who reported that the phage host range is not always restricted (El-Safty, 2021). A phage cocktail improves efficacy by increasing host coverage, decreasing resistance emergence, and enabling multi-receptor targeting and synergistic lysis, making it a more robust antibacterial strategy compared to monophage therapy (Yang et al., 2020). The phage cocktail’s narrow host range, restricted to *E. cloacae* with no activity against other bacterial species, is consistent with receptor-driven host tropism reported in other Enterobacteriaceae-infecting phages, where even minor variations in surface receptor structures among clinically related isolates can substantially restrict phage infectivity (Beamud et al., 2023).

Thermal stability analysis demonstrated that the isolated *E. cloacae* phage cocktail survived at all tested temperatures, from −20 to 50 °C, and that progressive titer reduction was observed upon exposure to temperatures between 55 °C and 70 °C, with complete inactivation achieved following 60 min of incubation at 90 °C. Our results in this regard are in accordance with those of a previous study by Jamal et al., who reported that phage MJ2 of *E. cloacae* was stable at temperatures ranging from 37 to 70 °C and lost its activity at 80 °C (Jamal et al., 2019). Bacteria can rapidly develop resistance to phage predation through mutations in receptor-encoding genes that phages use for adsorption. The application of phage cocktails targeting multiple bacterial surface receptors has been shown to effectively minimize the emergence of such resistance (Subedi et al., 2025). The observed resistance of the phage cocktail to chloroform treatment, with less than 1% reduction in titer, is consistent with the well-known chemical stability pattern of tailed bacteriophages within the class *Caudoviricetes*. Unlike membrane-containing phages, which possess an essential lipid bilayer and are therefore highly sensitive to chloroform, tailed phages have a purely proteinaceous capsid and tail structure devoid of lipid components, making them intrinsically resistant to lipid solvents such as chloroform (Mäntynen et al., 2019). This physicochemical robustness is also consistent with the requirements for downstream phage formulation and delivery in therapeutic applications (Malik et al., 2017).

Transmission electron microscopy imaging revealed that all four *Enterobacter cloacae*-infecting phages (MMRP1, MMRP2, MMRP3, and MMRP4) belong to the class *Caudoviricetes*, exhibiting icosahedral capsids, a tailed morphology, and double-stranded DNA genomes, consistent with current ICTV classification criteria (Turner et al., 2023). The phages identified were all lytic, which can be highly useful as therapeutic agents due to their strong structural integrity, lytic enzymatic activity, and effective mechanisms for delivering genetic material into host cells (Asif et al., 2021; Shi et al., 2018).

Whole-genome sequencing of the isolated MMRP1 and MMRP4 bacteriophages revealed a circular double-stranded DNA genome with large genome sizes (131,637 bp and 148,101 bp) for MMRP1 and MMRP4, respectively. The genome encodes a highly diverse and functionally organized repertoire of genes arranged in a characteristic modular architecture, a hallmark feature of the class of large-tailed *Caudoviricetes* phages and the *Siphoviridae* family, but cannot be considered to belong to the category of jumbo phages because it does not exceed the 200 kb threshold used to define such (Yutin et al., 2021; Zerbini et al., 2023). The structural gene modules of MMRP1 and MMRP4 encode key virion assembly and DNA packaging components, including the major head protein, portal protein, large terminase subunit, prohead protease, tail tube protein (pb6), tape measure protein (pb2), distal tail protein (pb9), and tail completion protein (p143). The presence of genes that encode such proteins in the genome maps of MMRP1 and MMRP4 is consistent with the morphological features reported for large Enterobacter-infecting phages (Kanhirun et al., 2024). Notably, the genome sizes of MMRP1 and MMRP4 fall within the range reported for recently characterized *Enterobacter cloacae* complex phages from Southern Thailand, which similarly exhibited modular genomes encoding structural, lytic, and replication gene clusters without evidence of resistance determinants (Yaikhan et al., 2025).

The lytic machinery of MMRP1 and MMRP4 is anchored by a holin gene that encodes a small membrane protein that governs the precise timing of host cell lysis by permeabilizing the cytoplasmic membrane. As a phylogenetically conserved component of the holin-endolysin system across tailed bacteriophages, burst size, and its identification in MMRP1 confirm the strict lytic character of the MMRP1 phage, with a robust bactericidal potential relevant to therapeutic applications (Khambhati et al., 2023). The replication and nucleotide metabolism modules of MMRP1 and MMRP4 encode key enzymes, including phage-associated DNA primase/helicase, DNA polymerase, thymidylate synthase, and ribonucleoside-diphosphate reductase, which confer substantial replicative autonomy from host nucleotide biosynthesis. Such replicative self-sufficiency has been documented in several *Enterobacter-* and *Klebsiella*-infecting phages with large genomes (Yutin et al., 2021).

A GC skew analysis performed across the MMRP1 and MMRP4 genes revealed alternating positive and negative skew regions distributed across the circular chromosome. This bimodal GC skew pattern is consistent with bidirectional replication initiated from a single origin of replication (oriC), as has been documented for numerous tailed bacteriophages with genome sizes in the 100–200 kbp range (Yuan and Gao, 2017).

The study revealed that the presence of a CRISPR-associated type IA (CAS-Type IA) cluster at a defined genomic locus of MMRP4. The identification of a phage-encoded CRISPR–Cas system is a finding of substantial biological and translational significance where it is proposed to function as a counter-defense mechanism against competing mobile genetic elements, bacterial restriction modification systems, or competing phages within the same host. The presence of a Type IA system in MMRP4 suggests that this phage may have acquired CRISPR-Cas machinery via horizontal gene transfer from a bacterial host or another co-infecting phage, a phenomenon consistent with the known mobilization of CRISPR elements between phage and bacterial genomes (Koonin and Makarova, 2019; Khambhati et al., 2023).

The phylogenetic analysis of the MMRP1-assembled genome helped to place the isolate in a well-supported monophyletic clade of characterized phages, namely the *Demereciviridae* family, which infects members of the family *Enterobacteriaceae*, such as *Klebsiella* and *Enterobacter* spp., which share common surface receptors that include lipopolysaccharide O-antigens and type IV pili. Phylogenomic studies identified MMRP4 as a member of the *Straboviridae* family, with the isolate exhibiting the strongest evolutionary relationship to characterized *Salmonella* and *Enterobacter* phages in this family. This phylogenetic tree indicates that although MMRP4 shares a common ancestor with these phages, it nevertheless represents a new genomic variant within the *Straboviridae* lineage. This degree of divergence is consistent with geographic and host-specific evolutionary forces that could act on phages isolated in nosocomial settings in Iraq, where specific bacterial populations and associated phageomes might diverge from those exemplified by phages stored in international reference databases (Adriaenssens and Brister, 2017).

The intergenomic similarity data generated by VIRIDIC (Moraru et al., 2020) provide robust genomic evidence for the taxonomic novelty of phages MMRP1 and MMRP4. Their shared affiliation with *Enterobacter* phage KKP-3711 at similarity values consistent with genus-level but not species-level relatedness, according to current ICTV criteria (Turner et al., 2023), suggests that these three phages may constitute founding members of a previously undescribed genus within the class *Caudoviricetes*. This finding is particularly notable given that *Kononvirus* KKP-3711 had not, until now, been reported to share substantial nucleotide identity with any other sequenced phage genome (Shymialevich et al., 2024), underscoring the value of continued phage isolation from clinical and environmental reservoirs in uncovering previously unrecognized viral diversity. This pattern of establishing novel taxonomic units based on VIRIDIC-derived intergenomic similarity and ICTV demarcation thresholds is consistent with recent reports characterizing other lytic phages of clinically relevant Gram-negative pathogens, such as *vB-Kpn-ZCKp20p* against multidrug-resistant *Klebsiella pneumoniae* (Zaki et al., 2023), further supporting the reliability of this approach for delineating novel phage genera and species. These phages represent the first reported isolates of this genomic lineage from Iraq and the Middle East, highlighting their regional epidemiological novelty.

Several limitations should be acknowledged: therapeutic efficacy was assessed only through in-vitro assays, and in-vivo validation is required before clinical translation. Additionally, statistical analyses were limited by the relatively small sample size, warranting larger-scale studies to confirm these findings.

## 5 Conclusion

Four new lytic bacteriophages, MMRP1, MMRP2, MMRP3, and MMRP4, targeting MDR, XDR, and PDR *E. cloacae* clinical isolates, were successfully isolated and fully characterized. The phage cocktail exhibited strong, selective lytic activity against 81.4% of clinical isolates, and its physicochemical stability was suitable for therapeutic use. Transmission electron microscopy indicated that all four isolated *E. cloacae*-infecting phages belong to the class *Caudoviricetes*. Definitive family-level classification of two phages (MMRP1 and MMRP4) into the evolutionary *Demereciviridae* and *Straboviridae* was achieved using whole-genome sequencing and comparative phylogenetic analysis, revealing that they harbored fully modular genomes with complete structural, lytic, and replication gene repertoires. The presence of a phage-encoded CRISPR-Cas Type IA cluster in the MMRP4 genome is of significant translational value and could potentially be used to design precision antimicrobials to combat resistance determinants in MDR pathogens.

Notably, the single phages MMRP1-MMRP4 exhibited high lytic activity against clinical MDR *E. cloacae* isolates. The most striking and highlighted suggestion that *in vitro* evaluation of MMRP1 and MMRP4 are highly recommended for deeper future experimental studies to combat MDR *E. cloacae* nosocomial infections, supported by genomic foundation, and eventually, the possibility to be suitable for phage-engineering applications in clinical settings. Within the global context of antimicrobial resistance, the findings broaden our existing knowledge of phage diversity and genomics and provide pertinent data for designing phage therapy as an alternative or supplementary tool to traditional antimicrobial-based approaches. Further, the study phages MMRP1 (GenBank accession PZ534072) and MMRP4 (GenBank accession PZ534073) are the first closely related relatives of Kononvirus KKP_3,711 reported to date, placing both phages within the same novel genus.

## Data Availability

The datasets presented in this study can be found in online repositories. The complete genome sequences of bacteriophages MMRP1 and MMRP4 have been deposited in GenBank under accession numbers PZ534072 (https://www.ncbi.nlm.nih.gov/nuccore/PZ534072) and PZ534073 (https://www.ncbi.nlm.nih.gov/nuccore/PZ534073), respectively. The corresponding BioSample records are available under accession numbers SAMN57629226 (https://www.ncbi.nlm.nih.gov/biosample/SAMN57629226) and SAMN57629227 (https://www.ncbi.nlm.nih.gov/biosample/SAMN57629227), under BioProject PRJNA1460573 (https://www.ncbi.nlm.nih.gov/bioproject/PRJNA1460573). Raw sequencing reads are available in the NCBI Sequence Read Archive (SRA) under accession numbers SRR38340615 for MMRP1 (https://trace.ncbi.nlm.nih.gov/Traces/index.html?run=SRR38340615) and SRR38340614 for MMRP4 (https://trace.ncbi.nlm.nih.gov/Traces/?run=SRR38340614). Any additional raw data supporting the conclusions of this article will be made available by the authors upon reasonable request.

## Supplementary material

Table S1 Functional gene annotation of the MMRP1 genome (Pharokka/PHANOTATE output).

Table S2 Functional gene annotation of the MMRP4 genome, including CRISPR-Cas Type IA cluster and holin (hol) gene annotation.

Figure S1 VIRIDIC intergenomic similarity heatmap/dendrogram for MMRP1 versus reference phage genomes.

Figure S2 VIRIDIC intergenomic similarity heatmap/dendrogram for MMRP4 versus reference phage genomes.

Figure S3 Agarose gel electrophoresis showing extracted genomic DNA of bacteriophages MMRP1 (lane 2) and MMRP4 (lane 3), confirming DNA integrity prior to whole-genome sequencing. Lanes 1, 4, and 5 correspond to negative control samples.

## References

[ref1] Adriaenssens E. M. Brister J. R. (2017). How to name and classify your phage: an informal guide. Viruses 9:70. doi: 10.3390/v9040070, 28368359 PMC5408676

[ref2] Al-Ouqaili M. T. Ahmad A. Jwair N. A. Al-Marzooq F. (2025). Harnessing bacterial immunity: CRISPR-Cas system as a versatile tool in combating pathogens and revolutionizing medicine. Front. Cell. Infect. Microbiol. 15:1588446. doi: 10.3389/fcimb.2025.1588446, 40521034 PMC12162490

[ref3] Alzaidi J. R. Mohammed A. S. (2022). First record of dissemination of BLBLI-resistant *Enterobacter cloacae* from public hospitals in Baghdad, Iraq. Open Microbiol. J. 16:e187428582201310. doi: 10.2174/18742858-v16-e2201310

[ref4] Asif M. Naseem H. Alvi I. A. Basit A. Rehman S. U. (2021). Characterization of a lytic EBP bacteriophage with a large size genome against *Enterobacter cloacae*. APMIS 129, 461–469. doi: 10.1111/apm.13138 33950561

[ref5] Beamud B. García-González N. Gómez-Ortega M. González-Candelas F. Domingo-Calap P. Sanjuan R. (2023). Genetic determinants of host tropism in Klebsiella phages. Cell Rep. 42:112048. doi: 10.1016/j.celrep.2023.112048, 36753420 PMC9989827

[ref6] Csiki-Fejer E. Traczewski M. Procop G. W. Davis T. E. Hackel M. Zambardi G. (2025). Performance evaluation of plazomicin susceptibility testing of Enterobacterales using VITEK 2 and VITEK 2 compact systems. J. Clin. Microbiol. 63, e00449–e00425. doi: 10.1128/jcm.00449-25 40748078 PMC12421896

[ref7] Daubie V. Chalhoub H. Blasdel B. Dahma H. Merabishvili M. Glonti T. . (2022). Determination of phage susceptibility as a clinical diagnostic tool: a routine perspective. Front. Cell. Infect. Microbiol. 12:1000721. doi: 10.3389/fcimb.2022.1000721, 36211951 PMC9532704

[ref8] El-Safty R. A. (2021). Isolation and characterization of soil-borne lytic bacteriophages infecting *Enterobacter* spp. causing fire blight-like disease on apple in Egypt. Al-Azhar Univers. J. Virus Res. Stud. doi: 10.21608/aujv.2021.199526

[ref9] Fu S.-Y. Chen X.-Z. Yi P.-C. Gao J. Wang W.-X. Gu S.-L. . (2025). Optimizing phage therapy for carbapenem-resistant *Enterobacter cloacae* bacteremia: insights into dose and timing. Antimicrob. Agents Chemother. 69:e01683–01624. doi: 10.1128/aac.01683-24, 40008877 PMC11963603

[ref10] Gonzalez-Gomez J. P. Rodriguez-Arellano S. N. Gomez-Gil B. de Jesús Vergara-Jiménez M. Chaidez C. (2024). Genomic and biological characterization of bacteriophages against *Enterobacter cloacae*, a high-priority pathogen. Virology 595:110100. doi: 10.1016/j.virol.2024.110100, 38714025

[ref11] Hyman P. (2019). Phages for phage therapy: isolation, characterization, and host range breadth. Pharmaceuticals (Basel) 12:35. doi: 10.3390/ph12010035, 30862020 PMC6469166

[ref12] Jamal M. Andleeb S. Jalil F. Imran M. Nawaz M. A. Hussain T. (2019). Das C. R. Das, C. R. (2019). Isolation, characterization and efficacy of phage MJ2 against biofilm-forming multi-drug resistant *Enterobacter cloacae*. Folia Microbiol., 64, 101–111. doi: DOI: 10.1007/s12223-018-0636-x 30090964

[ref13] Jwair N. A. Al-Ouqaili M. T. Al-Marzooq F. (2023). Inverse association between the existence of CRISPR/Cas systems and antibiotic resistance, extended spectrum β-lactamase and carbapenemase production in multidrug, extensive drug and pandrug-resistant *Klebsiella pneumoniae*. Antibiotics 12:980. doi: 10.3390/antibiotics12060980 37370299 PMC10295211

[ref14] Kąkol M. Tagliasacchi E. Borkowski A. Słowakiewicz M. (2023). Influence of different sample preparation techniques on imaging viruses and virus-like particles by scanning electron and scanning transmission electron microscopes. Front. Microbiol. 14:1279720. doi: 10.3389/fmicb.2023.1279720, 38033599 PMC10682772

[ref15] Kanhirun N. Blanc A. Aindow A. You M. Antani J. D. Ghatbale P. . (2024). Identification of a large cohort of *Enterobacter* jumbo phages with broad host ranges across pathogenic Gammaproteobacteria. [Epub ahead of print], 2024. doi:DOI: 10.1101/2024.10.29.620934

[ref16] Khambhati K. Bhattacharjee G. Gohil N. Dhanoa G. K. Sagona A. P. Mani I. . (2023). Phage engineering and phage-assisted CRISPR-Cas delivery to combat multidrug-resistant pathogens. Bioeng. Transl. Med. 8:e10381. doi: 10.1002/btm2.10381, 36925687 PMC10013820

[ref17] Kılıç G. Aybakan E. Tatlıoğlu N. Özgün G. Kocagöz T. Mozioğlu E. (2025). Simple, rapid, and efficient purification of M13 phages: Faj-Elek method. PLoS One 20:e0325621. doi: 10.1371/journal.pone.0325621 40478842 PMC12143533

[ref18] Koonin E. V. Makarova K. S. (2019). Origins and evolution of CRISPR-Cas systems. Philos. Trans. R. Soc. B Biol. Sci. 374:20180087. doi: 10.1098/rstb.2018.0087 PMC6452270 30905284

[ref19] Li Y. Liu S. Wang Y. Wang Y. Li S. He N. . (2023). Research on a magnetic separation-based rapid nucleic acid extraction system and its detection applications. Biosensors 13:903. doi: 10.3390/bios13100903 37887096 PMC10605191

[ref9020] Malik D. J. Sokolov I. J. Vinner G. K. Mancuso F. Cinquerrui S. Vladisavljevic G. T. . (2017). Adv. Colloid Interf. Sci. 249, 100–133. Advances in colloid and interface science, 249, 100–133. doi: 10.1016/j.cis.2017.05.014 28688779

[ref21] Manohar P. Tamhankar A. J. Lundborg C. S. Nachimuthu R. (2019). Therapeutic characterization and efficacy of bacteriophage cocktails infecting *Escherichia coli*, Klebsiella pneumoniae, and Enterobacter species. Front. Microbiol. 10:574. doi: 10.3389/fmicb.2019.00574, 30949158 PMC6437105

[ref22] Mäntynen S. Sundberg L.-R. Oksanen H. M. Poranen M. M. (2019). Half a century of research on membrane-containing bacteriophages: bringing new concepts to modern virology. Viruses 11:76. doi: 10.3390/v11010076, 30669250 PMC6356626

[ref23] Miller W. R. Arias C. A. (2024). ESKAPE pathogens: antimicrobial resistance, epidemiology, clinical impact and therapeutics. Nat. Rev. Microbiol. 22, 598–616. doi: 10.1038/s41579-024-01054-w, 38831030 PMC13147291

[ref24] Mohammadzadeh R. Shahbazi S. Khodaei N. Sabzi S. (2025). Emerging therapeutic strategies to combat antimicrobial resistance in the post-antibiotic era. J. Basic Microbiol. 65:e70070. doi: 10.1002/jobm.70070 40590508

[ref25] Moraru C. Varsani A. Kropinski A. M. (2020). VIRIDIC—A novel tool to calculate the intergenomic similarities of prokaryote-infecting viruses. Viruses 12:1268. doi: 10.3390/v12111268, 33172115 PMC7694805

[ref26] Mutai I. J. Juma A. A. Inyimili M. I. Nyachieo A. Nyamache A. K. (2022). Efficacy of diversely isolated lytic phages against multidrug-resistant *Enterobacter cloacae* isolates in Kenya. Afr. J. Labor. Med. 11:1673. doi: 10.4102/ajlm.v11i1.1673, 36091354 PMC9453119

[ref27] Sekhi A. H. Jaber A. S. (2025). Phenotypic characterization and virulence profiling of *Enterobacter* species isolated from clinical specimens in Thi-Qar Province, Iraq. Med. Sci. J. Adv. Res. 6. doi: 10.46966/msjar.v6i2.267

[ref28] Shakir S. M. Hussein N. H. Al-Kadmy I. M. Taha B. M. Hussein J. D. Batiha G. E.-S. . (2025). Exceptional prevalence of *blaNDM-*1 gene-producing *Enterobacter cloacae* ssp. *dissolvens* isolated from hospitals in Baghdad. Rev. Res. Med. Microbiol. 36, 80–87. doi: 10.1097/MRM.0000000000000384

[ref29] Shi H. Li J. Hao Y. Sun Y. (2018). Characterization of a novel lytic bacteriophage φEC14 that infects *Enterobacter cloacae* clinical isolates. Int. J. Bioautom. 22, 169–178. doi: 10.7546/ijba.2018.22.2.169-178

[ref30] Shymialevich D. Błażejak S. Średnicka P. Cieślak H. Ostrowska A. Sokołowska B. . (2024). Biological characterization and genomic analysis of three novel Serratia-and Enterobacter-specific virulent phages. Int. J. Mol. Sci. 25:5944. doi: 10.3390/ijms25115944, 38892136 PMC11172527

[ref31] Siaba S. Casal B. López-Martínez I. (2025). Economics of antibiotic resistance: a systematic review and meta-analysis based on global research: S. Siaba et al. Appl. Health Econ. Health Policy. 24, 15–46. doi: 10.1007/s40258-025-01001-7 40884690 PMC12790539

[ref32] Solanki R. Makwana N. Kumar R. Joshi M. Patel A. Bhatia D. . (2024). Nanomedicines as a cutting-edge solution to combat antimicrobial resistance. RSC Adv. 14, 33568–33586. doi: 10.1039/d4ra06117a/v1/review2 39439838 PMC11495475

[ref33] Subedi D. Gordillo Altamirano F. Deehan R. Perera A. Patwa R. Kostoulias X. . (2025). Rational design of a hospital-specific phage cocktail to treat *Enterobacter cloacae* complex infections. Nat. Microbiol. 10, 2702–2719. doi: 10.1038/s41564-025-02130-4 40993246 PMC12578640

[ref34] Tang K. W. K. Millar B. C. Moore J. E. (2023). Antimicrobial resistance (AMR). Br. J. Biomed. Sci. 80:11387. doi: 10.4060/cc6014en 37448857 PMC10336207

[ref35] Turner D. Shkoporov A. N. Lood C. Millard A. D. Dutilh B. E. Alfenas-Zerbini P. . (2023). Abolishment of morphology-based taxa and change to binomial species names: 2022 taxonomy update of the ICTV bacterial viruses subcommittee. Arch. Virol. 168:74. doi: 10.1007/s00705-022-05694-2, 36683075 PMC9868039

[ref36] Victoria-Blanco E. E. González-Gómez J. P. Medina-Sánchez J. R. Martínez A. A. Castro del Campo N. Chaidez-Quiroz C. . (2024). Characterization of *Enterobacter* phage vB_EcRAM-01, a new *Pseudotevenvirus* against *Enterobacter cloacae*, isolated from an urban river in Panama. PLoS One 19:e0310824. doi: 10.1371/journal.pone.0310824 39739645 PMC11687723

[ref37] Yaikhan T. Singkhamanan K. Luenglusontigit P. Chukamnerd A. Nokchan N. Chintakovid N. . (2025). Genomic analysis of *Enterobacter cloacae* complex from southern Thailand reveals insights into multidrug resistance genotypes and genetic diversity. Sci. Rep. 15:4670. doi: 10.1038/s41598-024-81595-5 39920182 PMC11806111

[ref38] Yang Y. Shen W. Zhong Q. Chen Q. He X. Baker J. L. . (2020). Development of a bacteriophage cocktail to constrain the emergence of phage-resistant *Pseudomonas aeruginosa*. Front. Microbiol. 11:327. doi: 10.3389/fmicb.2020.00327, 32194532 PMC7065532

[ref39] Yuan Y. Gao M. (2017). Jumbo bacteriophages: an overview. Front. Microbiol. 8:403. doi: 10.3389/fmicb.2017.00403, 28352259 PMC5348500

[ref40] Yutin N. Benler S. Shmakov S. A. Wolf Y. I. Tolstoy I. Rayko M. . (2021). Analysis of metagenome-assembled viral genomes from the human gut reveals diverse putative CrAss-like phages with unique genomic features. Nat. Commun. 12:1044. doi: 10.1038/s41467-021-21350-w 33594055 PMC7886860

[ref41] Zaki B. M. Fahmy N. A. Aziz R. K. Samir R. El-Shibiny A. (2023). Characterization and comprehensive genome analysis of novel bacteriophage, vB_Kpn_ZCKp20p, with lytic and anti-biofilm potential against clinical multidrug-resistant *Klebsiella pneumoniae*. Front. Cell. Infect. Microbiol. 13:1077995. doi: 10.3389/fcimb.2023.1077995, 36756618 PMC9901506

[ref42] Zerbini F. M. Siddell S. G. Lefkowitz E. J. Mushegian A. R. Adriaenssens E. M. Alfenas-Zerbini P. . (2023). Changes to virus taxonomy and the ICTV statutes ratified by the international committee on taxonomy of viruses (2023). Arch. Virol. 168:175. doi: 10.1007/s00705-024-06143-y 37296227 PMC10861154

[ref43] Zhu X. Yang K. Xie J. Feng X. Wu T. Hu M. . (2025). An SDS-NaOH-based method to isolate the genome of recombinant adeno-associated virus vectors for physical titer measurement. PLoS One 20:e0315921. doi: 10.1371/journal.pone.0315921 40179116 PMC11967980

