# Supplementary material for: Genomic and functional characterization of novel therapeutic lytic bacteriophages targeting multidrug-resistant Enterobacter cloacae
Source: Front Microbiol. 2026 Sep 1;17:1838809. doi: 10.3389/fmicb.2026.1838809 (PMC13574824; doi:10.3389/fmicb.2026.1838809)
Supplement: Supplementary file 1 [file Table_1.docx]

**Table 1. Complete open reading frames annotation of MMRP1 E. cloacae isolated bacteriophage**

| **MMRP1 E. cloacae isolated bacteriophage –** **Open Reading Frames Annotation** | | |
| --- | --- | --- |
| **Gene / Label** | **Predicted Function** | **Functional Category** |
| **Lysis** | | |
| L-alanyl-D-glutamate peptidase | Hydrolysis of the γ-D-glutamyl-meso-diaminopimelic acid bond in peptidoglycan; endolysin activity | Lysis |
| **Tail** | | |
| Distal tail protein pb9 | Baseplate distal tail component; host receptor binding and tail anchoring | Tail |
| Tape measure protein pb2 | Determines tail length; chaperone for tail fiber assembly | Tail |
| Tail tube protein pb6 | Forms the inner tail tube channel for DNA injection | Tail |
| Tail completion protein p143 | Baseplate hub wedge; completes tail assembly | Tail |
| **Coat** | | |
| Major head protein | Capsid structural subunit; icosahedral shell assembly | Coat |
| Protein A1 | Head–tail connector / adaptor structural protein | Coat |
| **Portal** | | |
| Portal protein | Dodecameric portal ring; DNA entry/exit channel | Portal |
| **Terminase** | | |
| Terminase, large subunit | ATPase/nuclease; headful DNA packaging | Terminase |
| **Replication / Metabolism** | | |
| DNA polymerase | Replicative DNA polymerase; phage genome replication | Replication / Metabolism |
| DNA primase / DNA helicase, phage-associated (copy 1) | Primer synthesis and DNA unwinding; lagging-strand replication | Replication / Metabolism |
| DNA primase / DNA helicase, phage-associated (copy 2) | Primer synthesis and DNA unwinding; lagging-strand replication (paralogue) | Replication / Metabolism |
| putative helicase D10 | Superfamily II helicase; DNA duplex unwinding | Replication / Metabolism |
| putative ssDNA-binding protein | Stabilizes ssDNA intermediates during replication | Replication / Metabolism |
| putative exonuclease subunit 2 | 5′→3′ exonuclease activity; DNA repair / recombination | Replication / Metabolism |
| Flap endonuclease | Removes 5′ flap structures; Okazaki-fragment maturation | Replication / Metabolism |
| Metallophos-2 domain-containing protein | Metal-dependent phosphohydrolase; nucleotide/nucleic-acid processing | Replication / Metabolism |
| Ribonucleotide-diphosphate reductase | Converts NDP to dNDP; deoxyribonucleotide biosynthesis | Replication / Metabolism |
| Anaerobic ribonucleoside triphosphate reductase | Anaerobic dNTP synthesis; class III reductase | Replication / Metabolism |
| Thymidylate synthase | dUMP → dTMP; thymidine biosynthesis | Replication / Metabolism |
| TPM domain-containing protein | Tail-to-phage membrane interaction; putative ejection protein | Replication / Metabolism |
