# Supplementary material for: Genomic and functional characterization of novel therapeutic lytic bacteriophages targeting multidrug-resistant Enterobacter cloacae
Source: Front Microbiol. 2026 Sep 1;17:1838809. doi: 10.3389/fmicb.2026.1838809 (PMC13574824; doi:10.3389/fmicb.2026.1838809)
Supplement: Supplementary file 2 [file Table_2.docx]

**Table 2. Complete open reading frames annotation of MMRP4 E. cloacae isolated bacteriophage**

| **MMRP4 E. cloacae isolated bacteriophage – Open Reading Frames Annotation** | | |
| --- | --- | --- |
| **Gene / Label** | **Predicted Function** | **Functional Category** |
| **Lysis** | | |
| hol | Holin; membrane channel formation triggering endolysin release at lysis | Lysis |
| **Tail protein** | | |
| Tail_protein (D16) | Baseplate/tail structural component; host adsorption | Tail protein |
| Tail_protein | Tail structural protein; tail tube/sheath component | Tail protein |
| Tail_protein | Tail structural protein (paralogue) | Tail protein |
| Tail_protein-D18 | Tail wedge/spike subunit; baseplate assembly | Tail protein |
| Tail_protein | Tail tube structural subunit | Tail protein |
| Tail_protein | Tail structural protein (paralogue) | Tail protein |
| mtp | Major tail protein; forms the tail tube polymer | Tail protein |
| ORF136 | Putative tail/baseplate structural protein (uncharacterised) | Tail protein |
| D20 | Baseplate hub structural protein | Tail protein |
| ORF144 | Putative tail structural ORF; function uncharacterised | Tail protein |
| Tail_protein (D17, pos. 1) | Tail spike / fibre subunit; receptor recognition | Tail protein |
| Tail_protein (D17, pos. 2) | Tail spike paralogue | Tail protein |
| Tail_protein (D17, pos. 3) | Tail spike paralogue | Tail protein |
| **Head protein** | | |
| Head_protein (Region-1) | Major capsid protein; icosahedral head assembly | Head protein |
| **Portal protein** | | |
| orf151 / Portal_protein | Portal ring protein; DNA encapsidation entry/exit | Portal protein |
| **Terminase** | | |
| A1 / Terminase | Large terminase subunit; headful DNA packaging ATPase | Terminase |
| **Fiber protein** | | |
| Fiber_protein (copy 1) | Tail fibre; primary host-surface receptor recognition | Fiber protein |
| Fiber_protein (copy 2) | Tail fibre paralogue; extended host range | Fiber protein |
| **Replication / Recombination / Repair** | | |
| pol | DNA polymerase; phage genome replication | Replication / Recombination / Repair |
| exo | Exonuclease; recombination-mediated DNA repair | Replication / Recombination / Repair |
| DNA_Helicase (D12) | ATP-dependent DNA helicase; duplex unwinding | Replication / Recombination / Repair |
| ligB | DNA ligase B; nick sealing in replication/repair | Replication / Recombination / Repair |
| td | Thymidylate synthase; dTMP biosynthesis | Replication / Recombination / Repair |
| **Endonuclease** | | |
| Endonuclease (D10) | Type II/IV endonuclease; DNA cleavage / restriction evasion | Endonuclease |
| Endonuclease (D11) | Endonuclease paralogue | Endonuclease |
| Endonuclease (Region-2) | Putative restriction endonuclease | Endonuclease |
| **Stability / Transfer / Defence** | | |
| CAS-TypeIA Cluster | CRISPR-Cas Type I-A system; phage-encoded anti-defence module | Stability / Transfer / Defence |
